# The emergence and spread of *bla*_NDM-1_, *bl*a_KPC-2_, *mcr-10* genes, and the *tmexCD2-toprJ2* gene cluster in extensively drug-resistant clinical *Raoultella ornithinolytica*

**DOI:** 10.3389/fcimb.2025.1675929

**Published:** 2025-11-05

**Authors:** Yafei Ye, Yuting Rao, Lei Fang, Haowei Ye, Ruyan Chen, Chenyu Li, Yanhao Shen, Yuanyuan Jia, Xiaobing Guo

**Affiliations:** ^1^ Department of Laboratory Medicine, The First Affiliated Hospital of Zhengzhou University, Zhengzhou, China; ^2^ Department of Laboratory Medicine of Puyang Oilfield General Hospital, Puyang, China; ^3^ State Key Laboratory for Diagnosis and Treatment of Infectious Diseases, National Clinical Research Center for Infectious Diseases, China-Singapore Belt and Road Joint Laboratory on Infection Research and Drug Development, National Medical Center for Infectious Diseases, Collaborative Innovation Center for Diagnosis and Treatment of Infectious Diseases, The First Affiliated Hospital, Zhejiang University School of Medicine, Hangzhou, China

**Keywords:** *Raoultella ornithinolytica*, *bla*_NDM-1_, *bla*_KPC-2_, *mcr-10*, *tmexCD2-toprJ2*

## Abstract

**Background:**

*Raoultella ornithinolytica* is an infrequent opportunistic pathogen capable of causing multi-site infections and frequently harboring a broad array of resistance determinants, thereby complicating antimicrobial therapy. Here we report the genomic characterization of the extensively drug-resistant strain FAHZZU6693, which concurrently harbors *bla*_NDM-1_, *bla*_KPC-2_, *mcr-10* genes and *tmexCD2-toprJ2* resistance cluster.

**Methods:**

Matrix-assisted laser desorption/ionization time-of-flight mass spectrometry (MALDI-TOF MS) and average nucleotide identity (ANI) were employed to confirm the species identity as *R. ornithinolytica*. Antimicrobial susceptibility testing (AST) delineated the corresponding antimicrobial phenotypes. S1 nuclease pulsed-field gel electrophoresis (S1-PFGE), Southern blotting and whole-genome sequencing (WGS) elucidated the isolate’s complete molecular architecture.

**Results:**

Globally, *R. ornithinolytica* strains harboring related resistance genes exhibit diverse geographical distribution. Strain FAHZZU6693 is resistant to most antibiotics, except amikacin and chloramphenicol. The *bla*_NDM-1_, *bla*_KPC-2_, *mcr-10* genes and the *tmexCD2-toprJ2* cluster in this strain are plasmid-borne. These occur in conserved genetic contexts: *xerC-mcr-10-*IS*Ec36-*IS*Ec27-*IS*Ecl1* and *umuC-*IS*881-tmexC2-tmexD2-toprJ2-umuC*. Further analysis indicates that the insertion sequence IS*Ec27* and the gene element *umuC* play a crucial role in the dissemination of the *mcr-10* gene and the *tmexCD2-toprJ2* gene cluster.

**Conclusions:**

This study combines database analysis to comprehensively describe the distribution of *R. ornithinolytica* strains carrying the target genes and characterizes the genomic features of a clinically Multi-drug resistant strain, providing a theoretical foundation for preventing the spread of such bacteria.

## 1 Introduction

*Raoultella ornithinolytica* is a Gram-negative, facultatively anaerobic bacillus possessing a capsule (De Coster et al., 2024). Molecular phylogeny based on *rpoB* sequencing led to its reclassification from the genus *Klebsiella* to *Raoultella* in 2001 (Etani et al., 2023). The organism is routinely recovered from water, soil and plants, and seafood has been identified as a potential reservoir of infection (Mader et al., 2013). Human isolates are documented sporadically, and clinical cases have been reported spanning the central nervous system, abdomen, eye, urinary tract, joints, biliary tract and bloodstream (Giran et al., 2025; Jones et al., 2024; Assadi et al., 2025).

In 2009, the first case of *R. ornithinolytica* carrying KPC-type carbapenemase causing an infection was reported in New Jersey, USA. The patient succumbed to the infection after failing to respond to anti-infective therapy. Antimicrobial susceptibility testing revealed that the strain was resistant to both imipenem and meropenem. Genomic sequencing demonstrated that the *bla*_KPC_ gene was located on a plasmid and was associated with the transposon Tn*4401c* (Castanheira et al., 2009). Four years later, the first case of *R. ornithinolytica* carrying NDM-1-type carbapenemase was reported in India. The strain caused a surgical site infection and exhibited resistance to imipenem and meropenem, as indicated by antimicrobial susceptibility testing. The patient recovered following treatment with tigecycline (Khajuria et al., 2013). Regrettably, no further investigation into the *bla*_NDM-1_ gene of this strain was conducted. Subsequently, in a *R. ornithinolytica* strain reported in China, the *bla*_NDM_ gene was found to be located within a Tn*125*-*like* element (IS*Aba125*-*bla*_NDM-1_-*ble*_MBL_-*trpF*-IS*Sen4*-Tn*5403*) and was situated on a transferable IncN-type incompatibility plasmid (Sun et al., 2015). In 2020, the first case of an *R. ornithinolytica* strain isolated from the environment that harbored both *bla*_NDM-1_ and *bla*_KPC-2_ was reported (Dang et al., 2020), indicating that different types of carbapenemase genes are continuously accumulating in *R. ornithinolytica*.

It was not until 2019 that the *mcr-8* gene was first reported in *R. ornithinolytica* from a poultry farm in China, with the strain harboring resistance to colistin (Wang et al., 2019). Additionally, in 2021, a *R. ornithinolytica* strain harboring the tigecycline resistance gene *tmexCD2-toprJ2* was first isolated from a sputum sample of a patient with a pulmonary infection. This strain was also positive for *bla*_NDM-1_ and *bla*_KPC-2_, exhibiting resistance to most antibiotics, including tigecycline, imipenem, and meropenem. Genomic studies revealed that the *tmexCD2-toprJ2* gene cluster is present on both the chromosome and an IncFI plasmid, and exists within a transposable unit (*xerD-like-int3-like-thf2-ybjD-umuD-ΔumuC1-int1-like-int2-like-hp1-hp2-tnfxB2-ISBvi2-tmexCD2-toprJ2-ΔumuC1*), confirming that this gene can be acquired through horizontal transfer (Wang et al., 2021).

Globally, the dissemination of tigecycline resistant *tmexCD-toprJ* and colistin resistant *mcr* genes among carbapenem-resistant *Enterobacterales* has precipitated extensively drug-resistant pathogens, prompting urgent clinical concern. Here we report the first isolation, from a blood culture, of *R. ornithinolytica* simultaneously harboring *bla*_NDM-1_, *bla*_KPC-2_, *mcr-10* and *tmexCD2-toprJ2* genes, all encoded on plasmids, conferring resistance to carbapenems, polymyxin B and tigecycline and posing a new challenge for antimicrobial therapy.

## 2 Materials and methods

### 2.1 Bacterial identification and antimicrobial susceptibility testing

In this study, bacterial species identification was performed using matrix-assisted laser desorption/ionization time-of-flight mass spectrometry (MALDI-TOF MS). PCR assays were conducted to detect the colistin resistance genes *mcr-1* to *mcr-10* and the tigecycline resistance gene cluster *tmexCD-toprJ*. Antimicrobial susceptibility testing (AST) was performed by broth microdilution for polymyxin B and tigecycline, and by agar dilution for all other agents. *Escherichia coli* ATCC 25922 was included for quality control. The breakpoints for tigecycline were interpreted according to the European Committee on Antimicrobial Susceptibility Testing (EUCAST) guidelines, while the other breakpoints were interpreted according to the Clinical and Laboratory Standards Institute (CLSI) guidelines (Humphries et al., 2021).

### 2.2 Plasmid characterization

The number and approximate size of plasmids in the isolate were determined by S1 nuclease–pulsed-field gel electrophoresis; Southern blotting confirmed that *bla*_NDM-1_, *bla*_KPC-2_, *mcr-10* and *tmexCD2-toprJ2* are plasmid-borne. The available recipient strains in the laboratory include rifampicin-resistant *Escherichia coli* EC600 and *Pseudomonas aeruginosa* PAORI, colistin-resistant *Pseudomonas aeruginosa* PAOCO, and azide-resistant *Escherichia coli* J53. However, due to the isolate’s resistance to rifampicin and colistin, as well as the intensified regulation on hazardous chemicals in China making sodium azide unavailable for laboratory verification, it’s impossible to use the existing recipient strains to confirm plasmid conjugation. Consequently, the experiment could only rely on oriTfinder for prediction.

### 2.3 Whole-genome sequencing and analysis

Genomic DNA was extracted using the SteadyPure Bacterial DNA Kit and sequenced on the Illumina NovaSeq 6000 (Illumina, San Diego, CA, USA) and Oxford Nanopore Technologies (Oxford, UK) platforms. Sequencing reads were assembled with Unicycler. Genome annotation was performed with Prokka. ResFinder was queried to identify acquired resistance determinants; plasmid types were assigned with PlasmidFinder. Insertion sequences and transposons were detected using ISFinder. Multiple plasmid sequences were compared with BLAST Ring Image Generator (BRIG), and genetic contexts of resistance genes were visualized in Easyfig. A phylogeny was constructed with Gubbins (Genealogies Unbiased By recomBinations In Nucleotide Sequences).

## 3 Results

### 3.1 Identification and resistance characterization of *R. ornithinolytica* FAHZZU6693

Strain FAHZZU6693 was isolated from a patient in the hematology department who was admitted for fever and mild pulmonary infection, later diagnosed with hemophagocytic lymphohistiocytosis. Despite etoposide (VP-16) treatment, the initial anti-infection regimen (biapenem) was ineffective, and the patient remained febrile. The antibiotic regimen was adjusted multiple times: piperacillin/tazobactam + voriconazole, piperacillin/tazobactam + voriconazole + imipenem, vancomycin + biapenem, tigecycline + piperacillin, and tigecycline + piperacillin + colistin. However, the therapeutic outcomes remained unsatisfactory, and the patient’s condition continued to deteriorate.

Strain FAHZZU6693 was identified as *R. ornithinolytica* by matrix-assisted laser desorption/ionization time-of-flight mass spectrometry (MALDI-TOF/MS). Its antimicrobial susceptibility profile is summarized in **Table 1**: the isolate was resistant to aztreonam, imipenem, meropenem, ceftriaxone, cefotaxime, ceftazidime, levofloxacin, ciprofloxacin, gentamicin, piperacillin-tazobactam, fosfomycin, trimethoprim-sulfamethoxazole, amoxicillin-clavulanate, cefepime, ceftazidime-avibactam, tigecycline and polymyxin B, but remained susceptible to amikacin and chloramphenicol.

**Table 1:** Antimicrobial susceptibility test results for FAHZZU6693 and control strain ATCC 25922.

Antimicrobials	MIC values (µg/mL )		Interpretive categories and MIC breakpoints, µg/mL		
	FAHZZU6693	ATCC25922	S	I	R
Aztreonam	64(R)	0.25(S)	≤4	8	≥16
Imipenem	16(R)	0.06(S)	≤1	2	≥4
Meropenem	16(R)	0.03(S)	≤1	2	≥4
Ceftriaxone	128(R)	0.125(S)	≤1	2	≥4
Cefotaxime	>128(R)	0.125(S)	≤1	2	≥4
Ceftazidime	>128(R)	0.5(S)	≤4	8	≥16
Levofloxacin	8(R)	0.03(S)	≤0.5	1	≥2
Ciprofloxacin	8(R)	<0.004(S)	≤0.25	0.5	≥1
Amikacin	2(S)	2(S)	≤4	8	≥16
Gentamicin	64(R)	1(S)	≤2	4	≥8
Piperacillin-tazobactam	>128(R)	2(S)	≤8/4	–	≥32/4
Fosfomycin	>512(R)	1(S)	≤64	128	≥256
Chloramphenicol	4(S)	8(S)	≤8	16	≥32
Trimethoprim-sulfamethoxazole	8(R)	≤0.125(S)	≤2/38	–	≥4/76
Amoxicillin-clavulanate	>128(R)	8(S)	≤8/4	16/8	≥32/16
Cefepime	16(R)	0.06(S)	≤2	–	≥16
Ceftazidime-avibactam	64(R)	0.25(S)	≤8/4	–	≥16/4
Tigecycline	2(R)	0.064(S)	≤1	–	≥2
Polymyxin B	4(R)	0.5(S)	–	≤2	≥4

### 3.2 Genomic characterization of *R. ornithinolytica* FAHZZU6693

*R. ornithinolytica* FAHZZU6693 contains a chromosome (5,475,585 bp) with an average GC content of 55.75%, two plasmids (1,917 bp and 4,172 bp), and three megaplasmids (127,231 bp, 263,150 bp, and 287,246 bp). PlasmidFinder assigned these to the incompatibility groups Col, Col440I, repB(R1701), IncFIB and IncU-repFIB-repHI5B, respectively. ResFinder identified 17 acquired resistance genes, located predominantly on the IncU-repFIB-repHI5B, IncFIB and repB(R1701) plasmids as well as on the chromosome; details are given in **Table 2**. OriTfinder results revealed that pFAHZZU6693-KPC-MCR and pFAHZZU6693-tmexCD-toprJ plasmids carry complete conjugal transfer modules that are capable of facilitating horizontal dissemination. These modules include the origin of transfer site (*oriT*), relaxase gene, genes encoding type IV coupling protein (T4CP), and genes encoding the bacterial type IV secretion system (T4SS). By contrast, pFAHZZU6693-NDM lacks the origin of transfer site (*oriT*).

**Table 2:** Genomic characteristics of *Raoultella ornithinolytica* FAHZZU6693.

Genome	Plasmids	Size (bp)	Antimicrobial resistance genes
Chromosome	NA	5,475,585	*bla*_PLA1a_, *fosA*
pFAHZZU6693-NDM	IncU-repFIB-repHI5B	287,246	*aadA16*, *aph(3')-Ia*, *aadA2*, *aac(6')-Ib*3, *bla*_NDM-1_, *qnrS1*, *arr-3*, *sul1*, *dfrA27*
pFAHZZU6693-KPC-MCR	IncFIB	263,150	*bla*_KPC-2_, *mcr-10*
pFAHZZU6693-tmexCD-toprJ	IncF(repB(R1701))	127,231	*tmexC2*, *tmexD2*, *toprJ2*
punamed1	Col440I	4,172	NA
punamed2	Col	1,917	NA

### 3.3 Phylogenetic analysis and multilocus sequence typing of *R. ornithinolytica*

**Figure 1:**
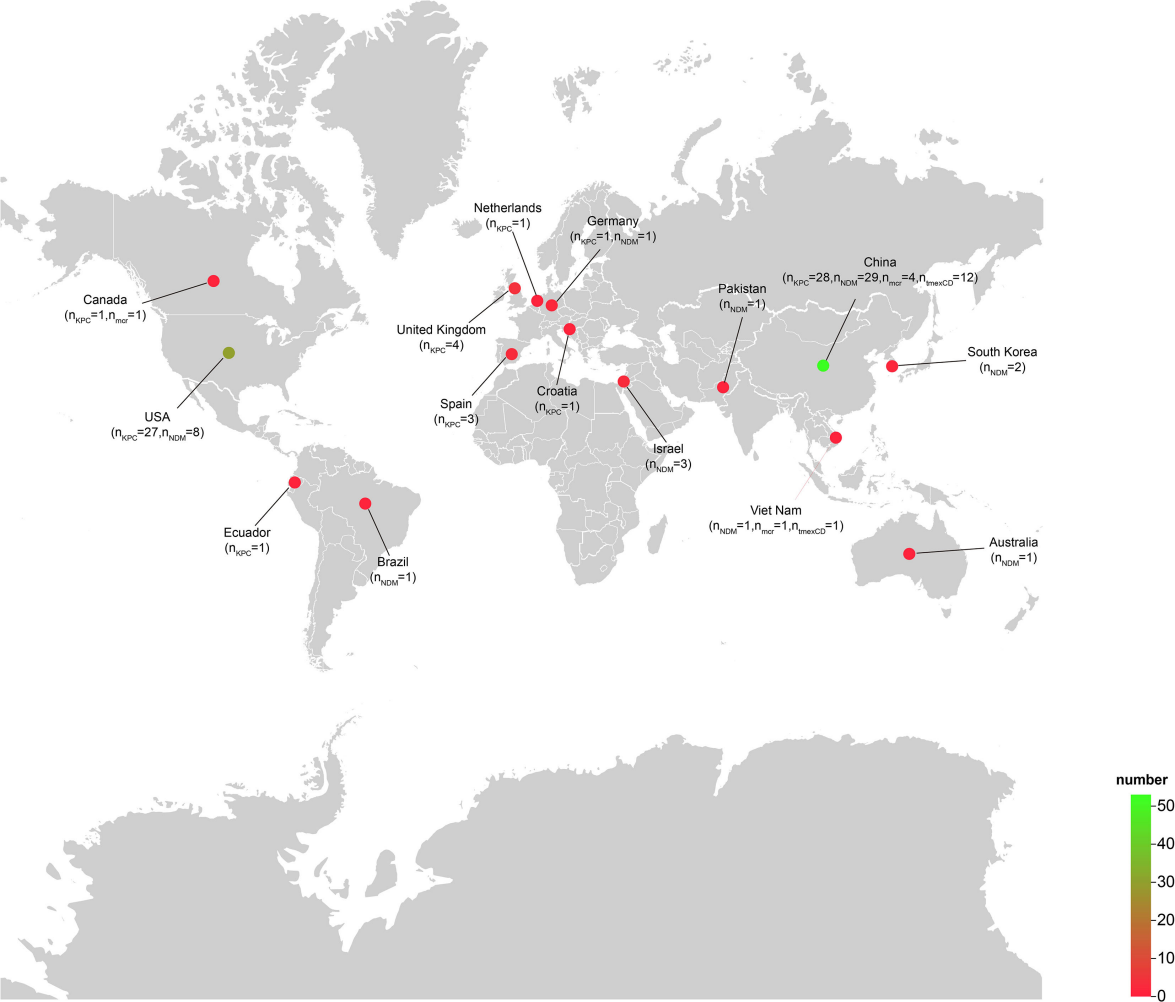
Geographical distribution of *Raoultella ornithinolytica* isolates harboring *bla*_NDM_, *bla*_KPC_, *mcr-10* or *tmexCD-toprJ*. A total of 106 *Raoultella ornithinolytica* genomes carrying at least one of the four resistance determinants were retrieved from NCBI; 105 of these had associated geographical metadata. The color gradient from red to green indicates an increasing number of isolates per country. The map was generated with the web platform ChiPlot (https://www.chiplot.online) (Xie et al., 2023).

A search of the NCBI database identified 586 *R. ornithinolytica* isolates. Resistance gene analysis revealed a detection rate of 11.3% for the *bla*_KPC_ gene, 8.2% for the *bla*_NDM_ gene, and 2.4% for isolates carrying both carbapenemase genes simultaneously. Additionally, the *mcr* gene, conferring colistin resistance, was detected in 1.0% of isolates, while the resistance-nodulation-division family (RND) efflux pump gene cluster *tmexCD-toprJ*, associated with tigecycline resistance, was identified in 2.2% of isolates. Notably, among *tmexCD-toprJ*-positive isolates, 46.1% co-harbored the *bla*_NDM_ gene, and 30.0% co-harbored colistin resistance genes (*mcr-10* or *mcr-8*). Further analysis showed that 106 of the 586 isolates carried at least one resistance determinant, including *bla*_KPC_, *bla*_NDM_, *tmexCD-toprJ*, or *mcr*. Of these, 1 isolate originated from animals, 60 from humans, and 45 from the environment. Strains carrying KPC or NDM-type carbapenemases were documented worldwide, with the majority recovered from China and the United States. In contrast, the *tmexCD-toprJ* gene cluster was only detected in isolates from China and Vietnam, indicating that Asia is the principal continental reservoir for *tmexCD-toprJ*-positive *R. ornithinolytica* (**Figure 1**).

**Figure 2:**
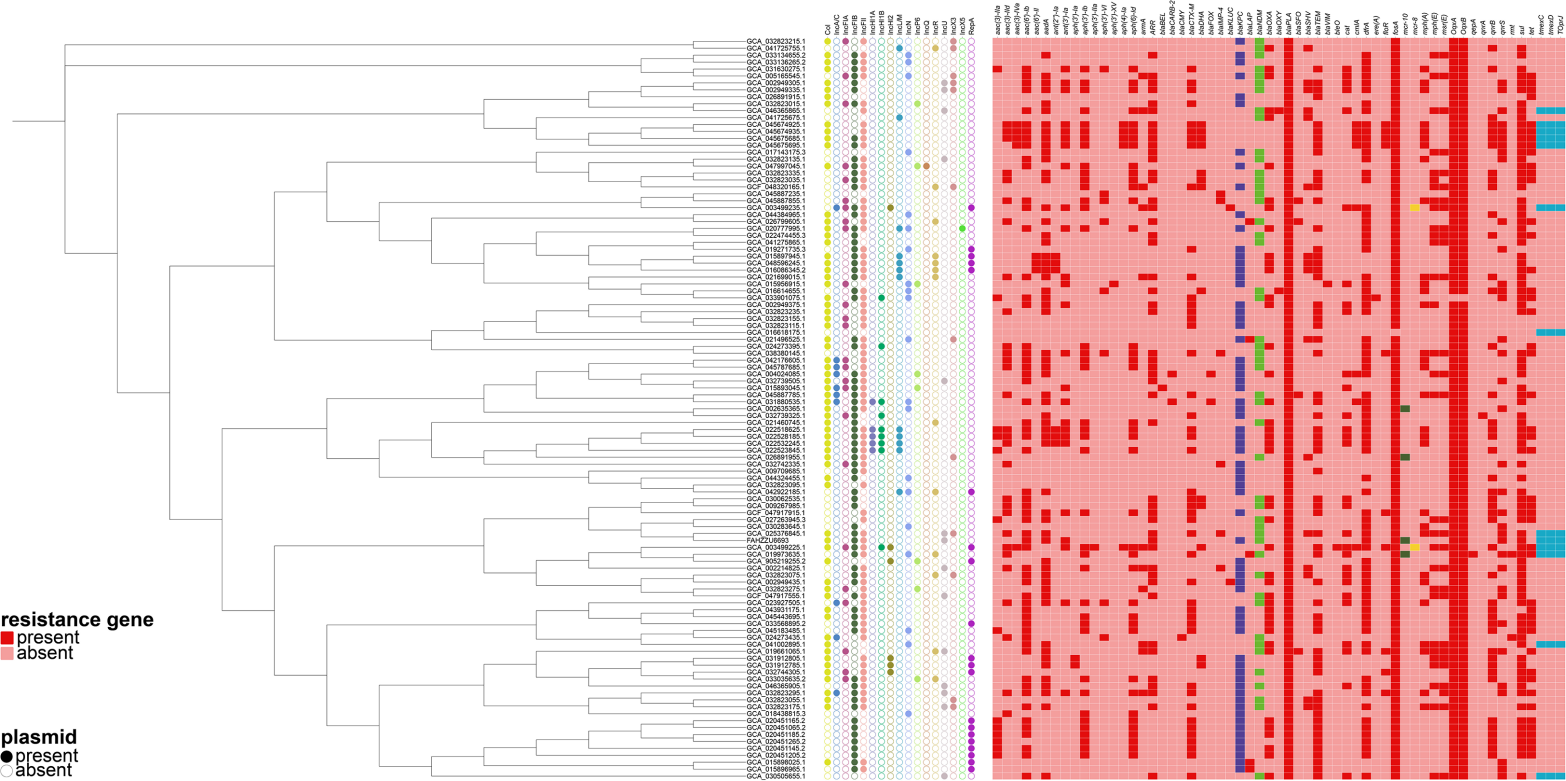
A phylogenetic tree was constructed based on the core genome sequences of the strain FAHZZU6693 used in this study and 106 *Raoultella ornithinolytica* strains selected from the National Center for Biotechnology Information (NCBI) database. A heatmap was then generated in conjunction with the plasmid types and antibiotic resistance genes carried by these strains.

Concurrently, we reconstructed a phylogeny that incorporated the 106 publicly available isolates together with strain FAHZZU6693 (**Figure 2**). The analysis identified GCA_025376845.1 as the closest evolutionary relative of FAHZZU6693; this isolate was recovered in 2019 from hospital sewage at the Second Affiliated Hospital of Soochow University, China. Compared with FAHZZU6693, it carries an additional IncX3 plasmid but lacks the resistance determinants *bla*_KPC-2_ and *mcr-10*. Across the entire collection, IncFII replicons were the most prevalent incompatibility group, followed by IncFIB. All strains harbored the β-lactamase gene *bla*_PLA_, and 99% additionally carried *fosA*, *oqxA* and *oqxB*. The *bla*_KPC_ was the dominant carbapenemase. Only a minority of isolates possessed the tigecycline-resistance determinant *tmexCD–toprJ* or the colistin resistance genes *mcr-10* and *mcr-8*.

Multilocus sequence typing (MLST) of strain FAHZZU6693 revealed a previously unassigned sequence type, defined by the allelic profile *gapA29*, *infB28*, *mdh63*, *pgi37*, *phoE47*, *rpoB28* and *tonB40*. Similarly, MLST of 106 selected *R. ornithinolytica* isolates showed that 105 represented unassigned sequence types, with *infB28*, *mdh63*, *pgi37*, *phoE7*, *rpoB43* and *tonB40* being the most prevalent alleles.

### 3.4 Structural characterization of the *bla*_NDM-1_-bearing plasmid

**Figure 3:**
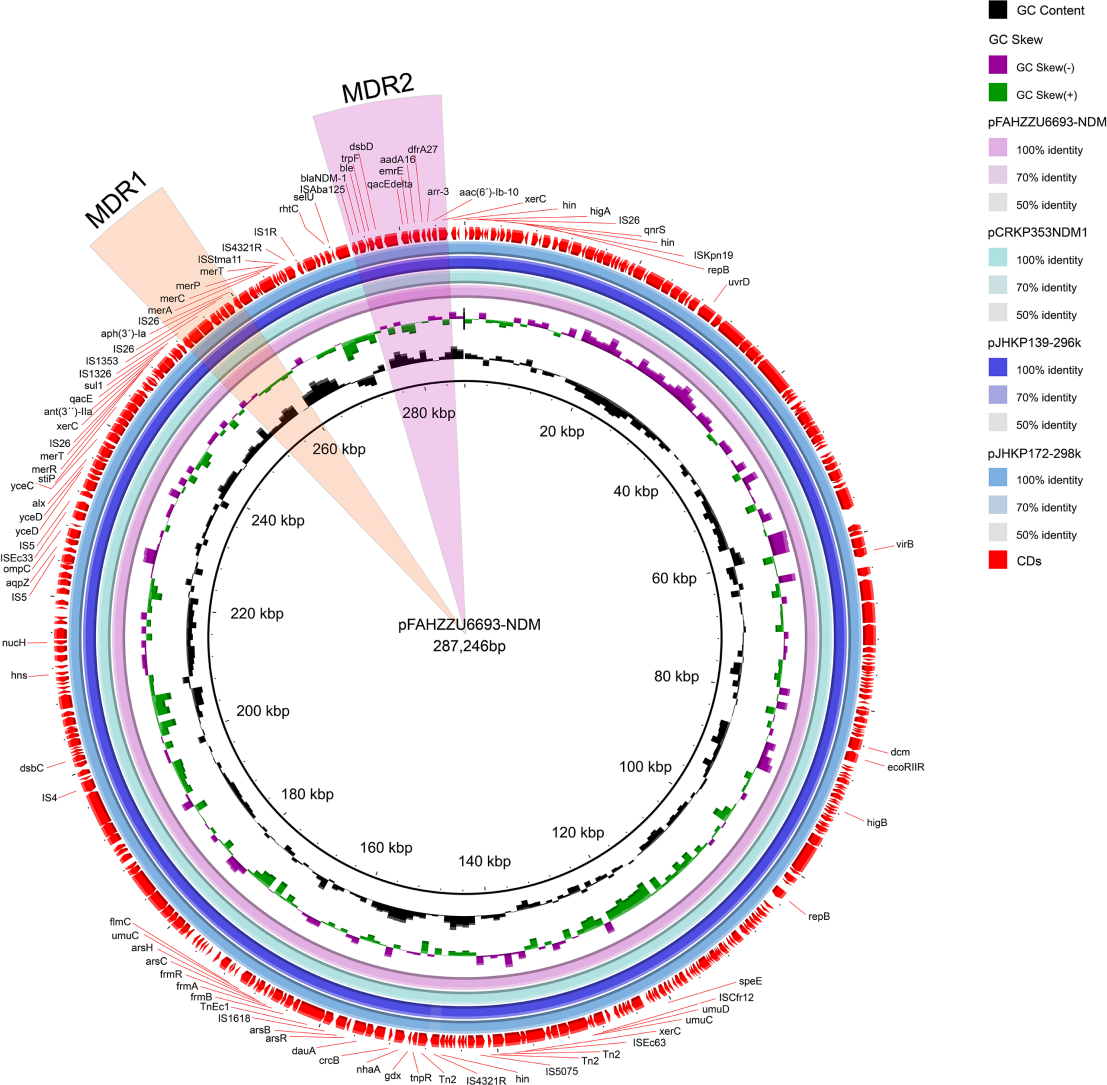
Circular map comparison of the pFAHZZU6693-NDM plasmid with the genomes of pCRKP353-NDM1, pJHKP139-296k, and pJHKP172-298k. The MDR1 and MDR2 regions are highlighted in orange and pink, respectively.

The carbapenem-resistance gene *bla*_NDM-1_ is harbored on the 287,246 bp IncU-repFIB-repHI5B plasmid pFAHZZU6693-NDM (47.7% GC). BLASTN searches of the NCBI nucleotide database revealed 100% query coverage and 100% nucleotide identity with pJHKP172-298k (CP180680.1) and pJHKP139-296k (CP180659.1) from *Klebsiella pneumoniae*, and 100% query coverage with 99.97% nucleotide identity to pCRKP353-NDM1 (CP141633.1) from *Klebsiella variicola* (**Figure 3**).This study demonstrates that pFAHZZU6693-NDM harbors two multidrug resistance (MDR) regions, designated MDR1 and MDR2. MDR1 spans 11,972 bp (coordinates 248,293–260,265 bp), is flanked by *xerC* and IS*26*, and encompasses a class 1 integron (248,293–250,700 bp) carrying the resistance gene *sul1*; within the integron are the aminoglycoside-resistance gene *ant(3´)-Ila* and the disinfectant-resistance gene *qacE*. MDR2 (coordinates 274,661–285,316; 10,655 bp) also contains a class 1 integron (280,812–285,316 bp) that harbors five resistance determinants. The carbapenemase gene *bla*_NDM-1_ is situated immediately upstream of this integron and confers resistance to carbapenems.

### 3.5 Structural characterization of co-harboring *bla*_KPC-2_ and *mcr-10* plasmid

**Figure 4:**
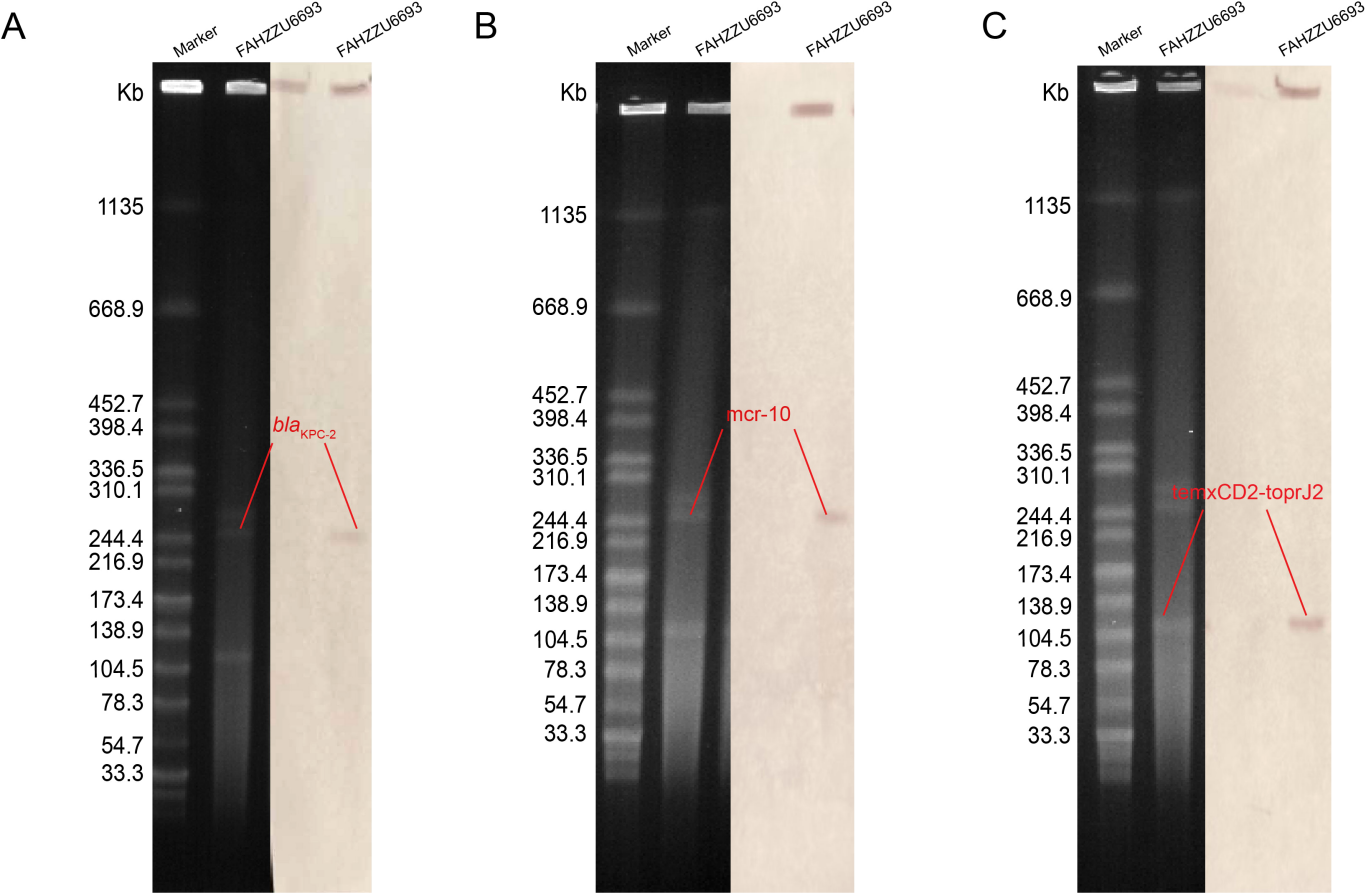
The S1 nuclease pulsed-field gel electrophoresis (S1-PFGE) profiles and Southern blot analysis results of strain FAHZZU6693 are shown. **(A–C)** illustrate the localization of the resistance genes *bla*_KPC-2_, *mcr-10*, and *tmcxCD2-toprJ2* to plasmids, respectively. The molecular weight marker used is *Salmonella enterica serovar* Braenderup H9812.

**Figure 5:**
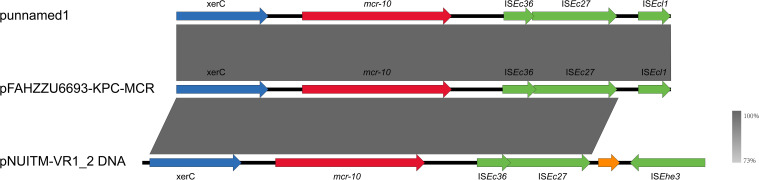
Genetic environment of the *mcr-10* gene in the pFAHZZU6693-KPC-MCR plasmid. Genes are represented in different colors: functional genes are shown in blue, the resistance gene *mcr-10* is highlighted in red, insertion elements are indicated in green, and hypothetical proteins are depicted in orange. The transcriptional direction of each gene is indicated by arrows.

S1-PFGE and Southern blotting, combined with whole-genome sequencing, revealed that the colistin resistance determinant *mcr-10* and the carbapenemase gene *bla*_KPC-2_ are co-located on the 52.19% GC IncFIB plasmid pFAHZZU6693-KPC-MCR (**Figures 4A, B**). To investigate the genetic context of *mcr-10* in *R. ornithinolytica*, we surveyed the NCBI repository and found three isolates carrying this colistin resistance determinant; whole-genome sequences were available for two. Comparative genomic analysis revealed that the *mcr-10* gene exhibits a conserved genetic structure of *xerC-mcr-10-*IS*Ec36-*IS*Ec27*. The *mcr-10* gene in punamed1, isolated from humans, and in FAHZZU6693-KPC-MCR shares an identical genetic context of *xerC-mcr-10-*IS*Ec36-*IS*Ec27-*IS*Ecl1*. In contrast, in the environmental plasmid pNUITM-VR1_2 DNA, the genetic environment of *mcr-10* is distinguished by IS*Ehe3* occupying the position of IS*Ecl1* (**Figure 5**). Additionally, PlasmidFinder assigned both punamed1 and pNUITM-VR1_2 DNA to the IncFIB–IncFII incompatibility group.

### 3.6 Structural characterization of the *tmexCD2-toprJ2*-harboring plasmid

**Figure 6:**
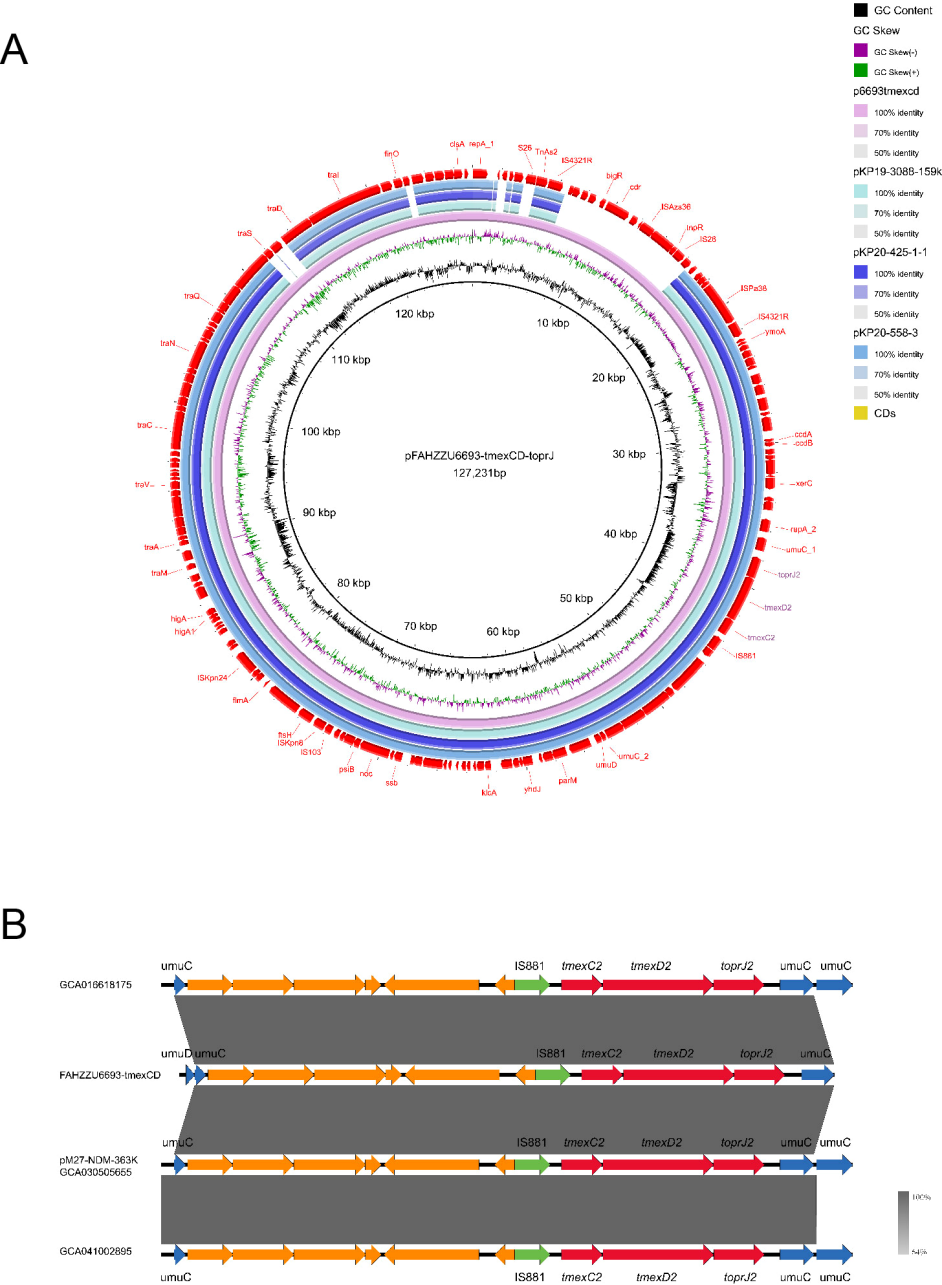
Genomic characteristics of the pFAHZZU6693-tmexCD-toprJ plasmid and the genetic environment of the *tmexCD2-toprJ2* gene cluster. **(A)** Circular map comparison of the pFAHZZU6693-tmexCD-toprJ plasmid with the genomes of pKP19-3088-159k, pKP20-425-1-1, and pKP20-558–3 plasmids. **(B)** Genetic environment of the *tmexCD2-toprJ2* gene cluster in *Raoultella ornithinolytica*. Genes are depicted in different colors: functional genes are shown in blue, the resistance gene *tmexCD2-toprJ2* is highlighted in red, insertion elements are indicated in green, and hypothetical proteins are shown in orange. The transcriptional direction of each gene is indicated by arrows.

The tigecycline resistance gene cluster *tmexCD2-toprJ2* is located on the IncF (repB(R1701)) plasmid pFAHZZU6693-*tmexCD-toprJ* (**Figure 4C**), which has a GC content of 53.83%. BLASTN searches in the NCBI nucleotide database revealed that pFAHZZU6693-tmexCD-toprJ shares 89% query coverage and over 99% nucleotide identity with plasmids from *R. ornithinolytica* pKP20-425-1-1 (accession number: CP109615.1) and *K. pneumoniae* pKP19-3088-159k and pKP20-558-3 (**Figure 6A**). To investigate the core genomic context of *tmexCD2-toprJ2* in *R. ornithinolytica*, we identified five *R. ornithinolytica* strains carrying *tmexCD2-toprJ2* through the NCBI database, of which three strains had whole-genome sequences. Among these, two strains harbored *tmexCD2-toprJ2* on the chromosome, while one strain carried it on a plasmid. The conserved genomic context of *tmexCD2-toprJ2* in *R. ornithinolytica* is *umuC*-IS*881*-*tmexC2-tmexD2-toprJ2-umuC* (**Figure 6B**). The presence of the insertion element IS*881* and flanking *umuC* genes endows these strains with the ability to acquire and mobilize the *tmexC2-tmexD2-toprJ2* cassette, thereby facilitating its dissemination among *R. ornithinolytica* strains.

## 4 Discussion

The coexistence of *bla*_NDM-1_, *bla*_KPC-2_, _mcr-10_, and *tmexCD2-toprJ2* genes in *R. ornithinolytica* is reported for the first time, with no prior reports documented. The strain was identified as Multi-drug resistant with an unknown sequence type (ST). Data analysis revealed that 99% of *R. ornithinolytica* strains lack defined ST, indicating that further research is needed for molecular typing of this species, which is of great significance for subsequent studies. Notably, 18.3% of *R. ornithinolytica* isolates were found to carry at least one carbapenemase, colistin, or tigecycline resistance gene, suggesting that this species has become a reservoir of multidrug resistance genes. More critically, the co-occurrence of *bla*_KPC_ and *bla*_NDM_ carbapenemase genes in the same strain may render most β-lactam antibiotics ineffective. Additionally, the high coexistence frequency of colistin and tigecycline resistance genes with carbapenemase genes may drive the emergence of “super-drug resistant” plasmids, thereby accelerating the spread of multidrug resistance. Sample sources indicate that *R. ornithinolytica* is not only widely present in human samples globally but has also been isolated from livestock and hospital wastewater, suggesting transmission pathways involving the environment, food, and healthcare settings. This underscores the importance of implementing integrated surveillance that encompasses human, animal, and environmental health within the framework of the “One Health” concept.

The whole-genome sequencing results of the FAHZZU6693 strain revealed that the *bla*_PLA_ and *fosA* genes are located on the chromosome, while all other resistance genes are harbored on plasmids. Analysis of the complete whole-genome sequences from the selected strains showed that all *R. ornithinolytica* strains carry the *bla*_PLA_ gene, which is located on the chromosome. This provides a genetic basis for the intrinsic resistance of *R. ornithinolytica* to amoxicillin, ampicillin, and ticarcillin. The study by Yang Lang et al. demonstrated that strains harboring two copies of the *bla*_NDM-1_ gene exhibit significantly enhanced resistance to carbapenems (Yang et al., 2021). The antimicrobial susceptibility profile of strain FAHZZU6693, which shows high resistance to imipenem and meropenem, is likely closely related to its co-carriage of both *bla*_NDM-1_ and *bla*_KPC-2_ genes. As previously reported, *K. pneumoniae* strains co-harboring *bla*_NDM_ and *bla*_KPC_ genes typically acquire a *bla*_NDM_-carrying plasmid in KPC-producing *K. pneumoniae (* Li et al., 2025). Similarly, considering the chronological order of reports on strains carrying different enzyme types, *R. ornithinolytica* strains harboring both *bla*_NDM-1_ and *bla*_KPC-2_ likely follow a similar acquisition pathway. Previous studies have confirmed that the *bla*_NDM_ gene can spread resistance gene among different bacterial species via plasmid-mediated horizontal transfer. In this study, analysis using OriTfinder revealed that the pFAHZZU6693-NDM plasmid carries a relaxase gene, a gene encoding a type IV coupling protein (T4CP), and a gene cluster for a bacterial type IV secretion system (T4SS); however, no origin of transfer site (*oriT*) was identified. Theoretically, the absence of *oriT* may limit the horizontal transfer capacity of this plasmid. However, prior research has indicated that most conjugative and mobilizable plasmids lack recognizable *oriT* sequences (Ares-Arroyo et al., 2024). The conjugal transfer ability of this plasmid has not yet been verified in this study, which represents one of its limitations. In future research, if sodium azide becomes available, we plan to further validate the transferability of pFAHZZU6693-NDM using the recipient strain J53.

To date, ten different *mcr* genes, including *mcr-1* to *mcr-10*, have been reported globally (Partridge et al., 2018). The *mcr-1* gene is the most common and widely distributed colistin resistance gene (Abdullah et al., 2024). Interestingly, the results of this study indicate that *mcr-10* has emerged as the most prevalent colistin resistance gene in *R. ornithinolytica*, followed by *mcr-8*. No other *mcr* gene variants have been reported in this species. This suggests that *mcr-10* may have acquired an adaptive advantage within *R. ornithinolytica*, potentially attributable to the conserved genetic structure of *xerC-mcr-10-*IS*Ec36-*IS*Ec27*. In this study, we observed that genetic sequence differences between environmental and human-derived strains are confined solely to the insertion sequence region downstream of IS*Ec27*. Furthermore, this study is the first to report the co-localization of *mcr-10* and *bla*_KPC-2_ on the same plasmid. Considering the large prevalence of carbapenem-resistant strains producing KPC-type carbapenemases in the region and the predominance of IncFIB-type plasmids (Mohamed et al., 2019), we hypothesize that in *R. ornithinolytica*, IS*Ec27* may mediate the horizontal transfer of the *mcr-10* gene on *bla*_KPC_-carrying IncFIB plasmids. Given that plasmids are capable of autonomous replication and horizontal transfer (Liu et al., 2024), this significantly increases the potential risk of the emergence of multidrug-resistant strains. Previous research has established a certain degree of biological relatedness between *R. ornithinolytica* and *K. pneumoniae*, with their genes exhibiting robust horizontal transfer capabilities (Zhai et al., 2025). Thus, the extensive drug resistance background already present in *K. pneumoniae* will further exacerbate the clinical infection risk associated with this pathogen.

Most concerning is the coexistence of the tigecycline resistance gene cluster *tmexCD2-toprJ2* and the colistin resistance gene *mcr-10* within the same strain, effectively breaching the last two lines of defense available in antibiotic selection (Cheng et al., 2020). A strain of *K. pneumoniae* isolated from a patient with pulmonary infection in China was found to harbor the *tmexC2-tmexD2-toprJ2* gene cluster on both the chromosome and a plasmid (Wang et al., 2021). This suggests that the *tmexCD2-toprJ2* gene cluster is capable of horizontal transfer between the chromosome and plasmid. The *tmexCD2-toprJ2* gene cluster exists in the structure of *umuC-*IS*881-tmexC2-tmexD2-toprJ2-umuC*, with 100% nucleotide identity, regardless of its location on the chromosome or plasmid. Previous studies have demonstrated that *umuC* has the capacity for DNA cleavage and repair (Murison et al., 2017), and it is widely distributed in genetic material. Thus, the *umuC* gene is likely to facilitate the horizontal transfer of the *tmexC2-tmexD2-toprJ2* structure. Given this, it is essential to further investigate the potential mechanisms of *umuC*-mediated gene transfer in order to curb the accumulation and dissemination of resistance genes among strains. Experimental research by Hong Yao et al. has demonstrated that IncF [repB(R1701)] has conjugative transfer capabilities. In terms of genetic structure, it contains both IncFII and IncFI replicons (Yao et al., 2024). Investigations into the plasmid types carried by *R. ornithinolytica* have shown that IncFII and IncFI plasmids are the two most common types. This provides a dual advantage for the dissemination of the *tmexC2-tmexD2-toprJ2* structure.

## 5 Conclusion

In this study, for the first time, the coexistence of *bla*_NDM-1_, *bla*_KPC-2_, *mcr-10*, and the *tmexC2-tmexD2-toprJ2* gene cluster was identified in a clinical isolate of *R. ornithinolytica*, with these genetic elements residing on plasmids. Comprehensive analysis of *R. ornithinolytica* strains from the NCBI database revealed distinct geographical distribution patterns for these resistance genes. The conserved genetic contexts of these elements, coupled with the presence of abundant plasmid-associated mobile genetic elements, suggest the enhanced acquisition and recombination of these resistance genes. *R. ornithinolytica*, as a reservoir of multidrug resistance genes, introduces a severe potential risk to public health. Thus, enhanced surveillance of this pathogen, particularly those strains harboring multiple resistance genes and genetic clusters, is of critical importance.

## Data Availability

The datasets presented in this study can be found in online repositories. The names of the repository/repositories and accession number(s) can be found below: https://www.ncbi.nlm.nih.gov/, PRJNA1267968.
