# Supplementary figures and images for: The emergence and spread of bla NDM-1, blaKPC-2, mcr-10 genes, and the tmexCD2-toprJ2 gene cluster in extensively drug-resistant clinical Raoultella ornithinolytica
Source: Front Cell Infect Microbiol. 2025 Nov 5;15:1675929. doi: 10.3389/fcimb.2025.1675929 (PMC12627040; doi:10.3389/fcimb.2025.1675929)

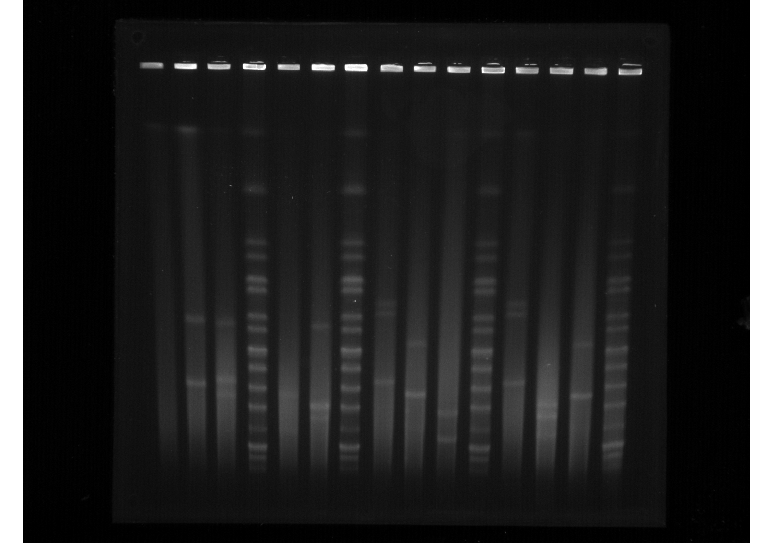

Supplement: Supplementary file 3 [file DataSheet1.zip › Original Blot and Gel Images/Original Gel Image 1.tif]

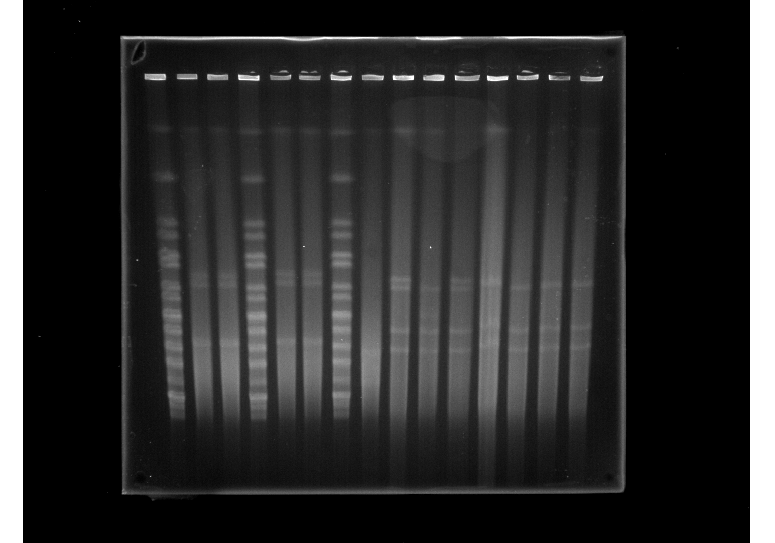

Supplement: Supplementary file 3 [file DataSheet1.zip › Original Blot and Gel Images/Original Gel Image 2.tif]

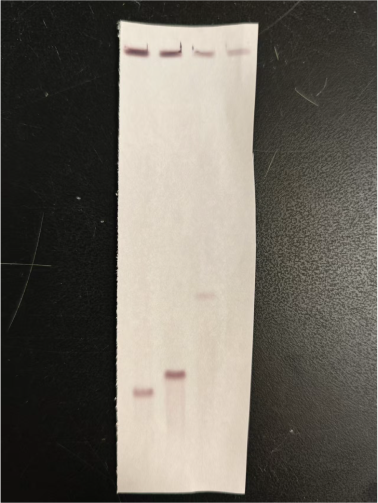

Supplement: Supplementary file 3 [file DataSheet1.zip › Original Blot and Gel Images/Original Image of blaKPC-2 Gene Blot.tif]

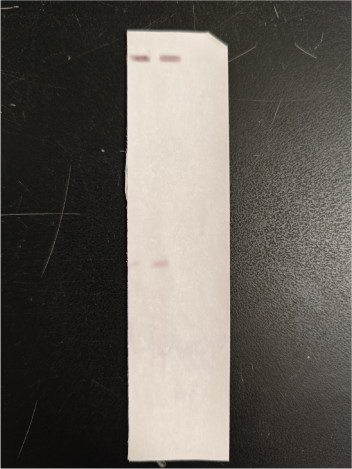

Supplement: Supplementary file 3 [file DataSheet1.zip › Original Blot and Gel Images/Original Image of mcr-10 Gene Blot.tif]

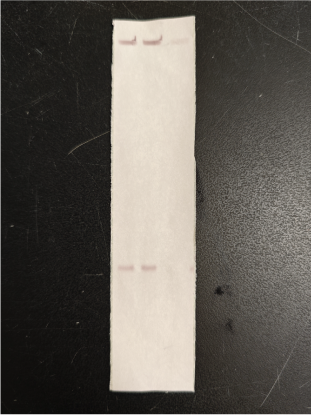

Supplement: Supplementary file 3 [file DataSheet1.zip › Original Blot and Gel Images/Original Image of tmexCD2-toprJ2 Gene Blot.tif]

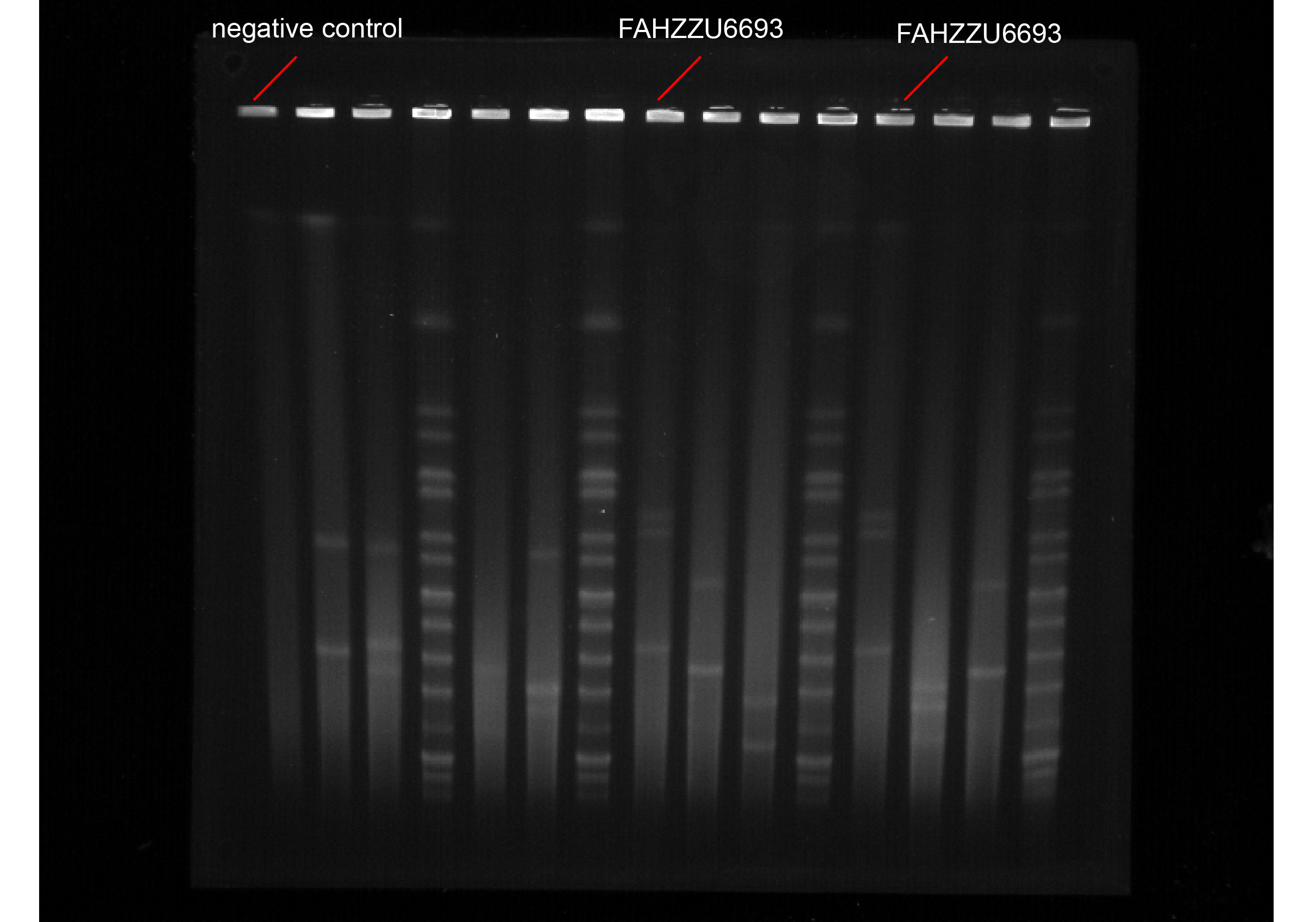

Supplement: Supplementary file 4 [file Image1.jpeg]

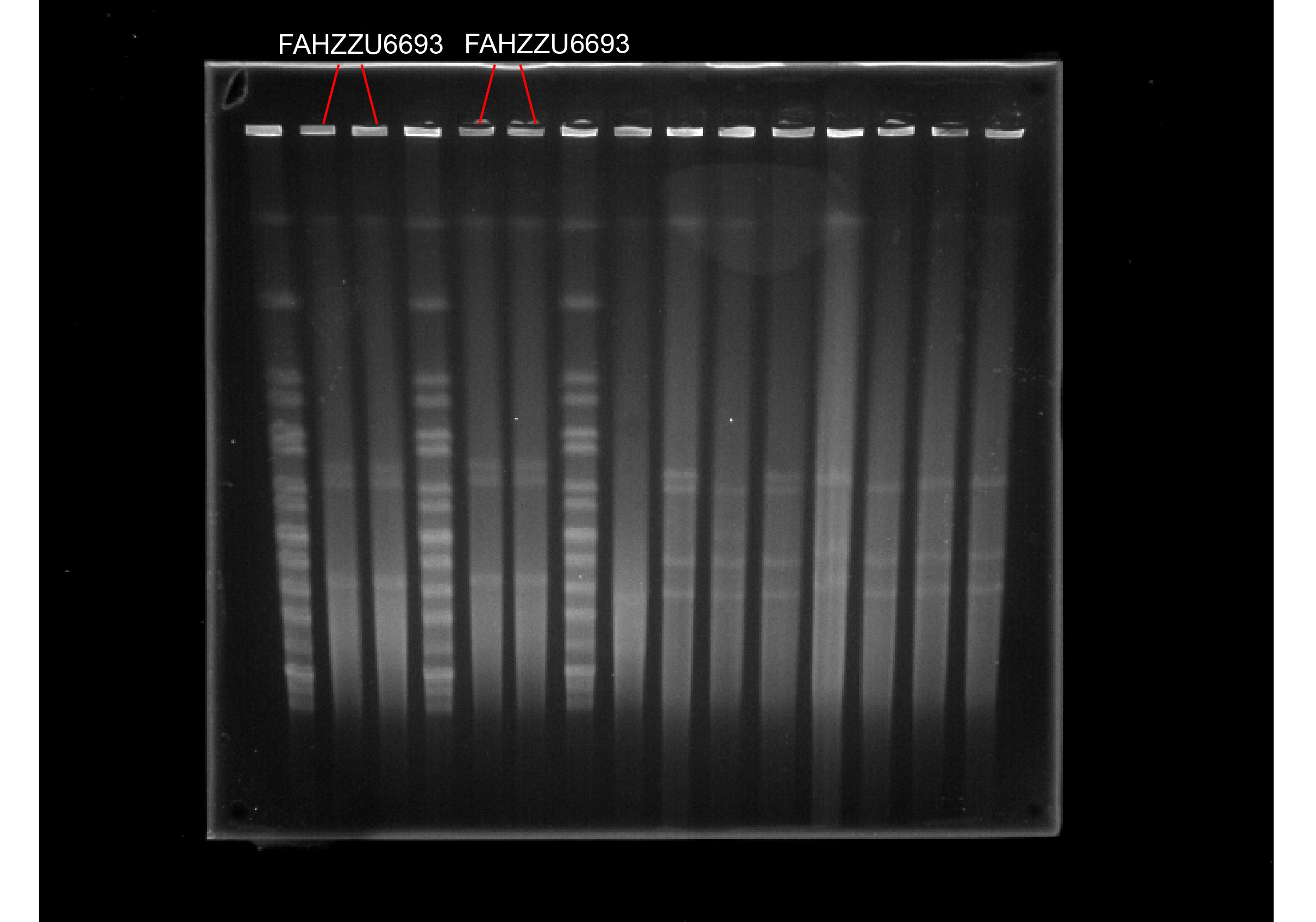

Supplement: Supplementary file 5 [file Image2.jpeg]
